# Incorporating clinical parameters to improve the accuracy of angiography-derived computed fractional flow reserve^[Author-notes ztac045-FM1]^

**DOI:** 10.1093/ehjdh/ztac045

**Published:** 2022-09-05

**Authors:** Rebecca C Gosling, Eleanor Gunn, Hua Liang Wei, Yuanlin Gu, Vignesh Rammohan, Timothy Hughes, David Rodney Hose, Patricia V Lawford, Julian P Gunn, Paul D Morris

**Affiliations:** Department of Infection, Immunity and Cardiovascular Disease, Medical School, Beech Hill Road, Sheffield, S102TN, UK; Department of Cardiology, Sheffield Teaching Hospitals NHS Foundation Trust, Herries Road, Sheffield, S57AU, UK; Insigneo institute for in silico medicine, Pam Liversidge building, Sheffield, S1 3JD, UK; Department of Infection, Immunity and Cardiovascular Disease, Medical School, Beech Hill Road, Sheffield, S102TN, UK; Department of Infection, Immunity and Cardiovascular Disease, Medical School, Beech Hill Road, Sheffield, S102TN, UK; Insigneo institute for in silico medicine, Pam Liversidge building, Sheffield, S1 3JD, UK; Department of Infection, Immunity and Cardiovascular Disease, Medical School, Beech Hill Road, Sheffield, S102TN, UK; Insigneo institute for in silico medicine, Pam Liversidge building, Sheffield, S1 3JD, UK; Department of Infection, Immunity and Cardiovascular Disease, Medical School, Beech Hill Road, Sheffield, S102TN, UK; Insigneo institute for in silico medicine, Pam Liversidge building, Sheffield, S1 3JD, UK; Department of Cardiology, Sheffield Teaching Hospitals NHS Foundation Trust, Herries Road, Sheffield, S57AU, UK; Department of Infection, Immunity and Cardiovascular Disease, Medical School, Beech Hill Road, Sheffield, S102TN, UK; Department of Cardiology, Sheffield Teaching Hospitals NHS Foundation Trust, Herries Road, Sheffield, S57AU, UK; Insigneo institute for in silico medicine, Pam Liversidge building, Sheffield, S1 3JD, UK; Department of Infection, Immunity and Cardiovascular Disease, Medical School, Beech Hill Road, Sheffield, S102TN, UK; Department of Cardiology, Sheffield Teaching Hospitals NHS Foundation Trust, Herries Road, Sheffield, S57AU, UK; Insigneo institute for in silico medicine, Pam Liversidge building, Sheffield, S1 3JD, UK; Department of Infection, Immunity and Cardiovascular Disease, Medical School, Beech Hill Road, Sheffield, S102TN, UK; Department of Cardiology, Sheffield Teaching Hospitals NHS Foundation Trust, Herries Road, Sheffield, S57AU, UK; Insigneo institute for in silico medicine, Pam Liversidge building, Sheffield, S1 3JD, UK; Department of Infection, Immunity and Cardiovascular Disease, Medical School, Beech Hill Road, Sheffield, S102TN, UK; Department of Cardiology, Sheffield Teaching Hospitals NHS Foundation Trust, Herries Road, Sheffield, S57AU, UK; Insigneo institute for in silico medicine, Pam Liversidge building, Sheffield, S1 3JD, UK

**Keywords:** Stable angina, Fractional flow reserve, Transthoracic echocardiogram

## Abstract

**Aims:**

Angiography-derived fractional flow reserve (angio-FFR) permits physiological lesion assessment without the need for an invasive pressure wire or induction of hyperaemia. However, accuracy is limited by assumptions made when defining the distal boundary, namely coronary microvascular resistance (CMVR). We sought to determine whether machine learning (ML) techniques could provide a patient-specific estimate of CMVR and therefore improve the accuracy of angio-FFR.

**Methods and results:**

Patients with chronic coronary syndromes underwent coronary angiography with FFR assessment. Vessel-specific CMVR was computed using a three-dimensional computational fluid dynamics simulation with invasively measured proximal and distal pressures applied as boundary conditions. Predictive models were created using non-linear autoregressive moving average with exogenous input (NARMAX) modelling with computed CMVR as the dependent variable. Angio-FFR (VIRTUheart™) was computed using previously described methods. Three simulations were run: using a generic CMVR value (Model A); using ML-predicted CMVR based upon simple clinical data (Model B); and using ML-predicted CMVR also incorporating echocardiographic data (Model C). The diagnostic (FFR ≤ or >0.80) and absolute accuracies of these models were compared. Eighty-four patients underwent coronary angiography with FFR assessment in 157 vessels. The mean measured FFR was 0.79 (±0.15). The diagnostic and absolute accuracies of each personalized model were: (A) 73% and ±0.10; (B) 81% and ±0.07; and (C) 89% and ±0.05, *P* < 0.001.

**Conclusion:**

The accuracy of angio-FFR was dependent in part upon CMVR estimation. Personalization of CMVR from standard clinical data resulted in a significant reduction in angio-FFR error.

## Graphical Abstract

**Graphical Abstract:**
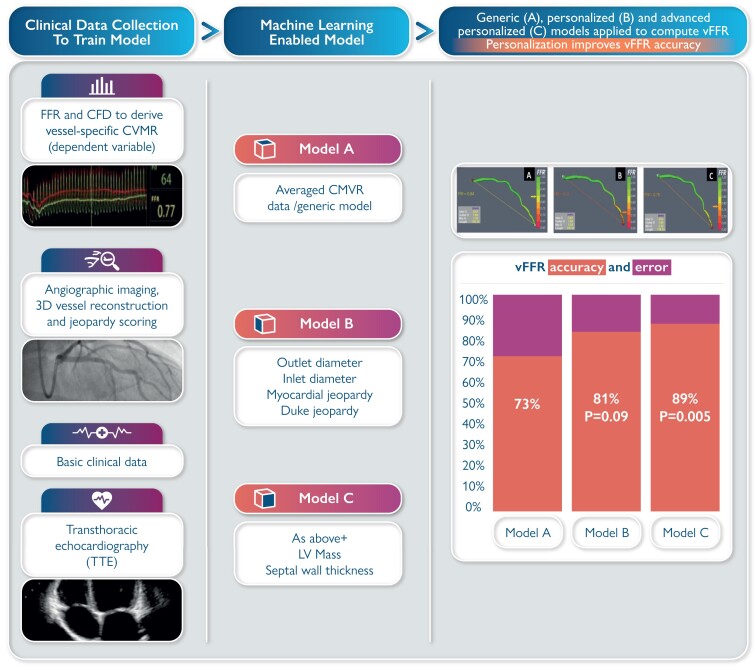
Clinical data and machine learning were utilized to improve the accuracy of angio-FFR. Data were collected from patients with chronic coronary syndromes and undergoing coronary angiography. Fractional flow reserve assessment, combined with computational fluid dynamics modelling, was utilized to determine the vessel-specific coronary microvascular resistance (CMVR). Other clinical and transthoracic echocardiographic (TTE) data were entered into a machine learning model to identify predictors of CMVR. Two models were created, the first incorporating basic clinical data (Model B) and the second incorporating TTE data. Model-predicted CMVR was then incorporated in vFFR computation. Both personalized models were compared with the standard vFFR method (Model A). Diagnostic accuracy increased on account of model personalization (Model A = 73%, Model B = 81%, Model C = 89%). CFD, computational fluid dynamics; CMVR, coronary microvascular resistance; FFR, fractional flow reserve; vFFR, virtual (angio-)fractional flow reserve.

## Introduction

Fractional flow reserve (FFR) is the gold standard method for determining physiological coronary artery lesion significance and guiding percutaneous coronary intervention (PCI).^1^ However, it remains underused due to practical and methodological constraints. Angiography-derived virtual fractional flow reserve (angio-FFR) permits the assessment of coronary physiology without the need for an invasive pressure wire or induction of hyperaemia.^2–4^ Typically, angio-FFR is calculated by applying a mathematical solution of flow, based upon the laws of fluid dynamics, to a geometric reconstruction of coronary anatomy, derived from the angiogram. A number of models have been developed, demonstrating reasonable diagnostic accuracy, i.e. the ability to predict whether FFR is  ≤ or >0.80. However, significant quantitative (absolute) errors have been reported, with 95% limits of agreement of FFR ±0.14 for most published models.^5^ One of the major sources of error relates to assumptions made when defining the boundary conditions of the model, and those of the distal boundary are particularly important.^6^ The distal boundary of a physiological coronary model represents the distal coronary microvascular resistance (CMVR), which is an important determinant of coronary blood flow. Without invasive measurement, the CMVR is unknown and so a generic value usually based on population averages data is typically applied. This is known to limit the accuracy of angio-FFR prediction. We hypothesized that lesion-specific CMVR could be predicted from patient data and that this would increase the accuracy of angio-FFR.

## Methods

### Study population

Patients with chronic coronary syndromes were recruited. Patients were excluded if they had presented acutely within the previous 60 days, had undergone previous coronary artery bypass graft surgery, had chronic total occlusion(s), if they were unable to consent or if was deemed dangerous to pass a pressure wire. All patients provided written informed consent. The study was approved by the local ethics committee (13/YH/0070). Clinical, demographic, FFR, angiographic, and echocardiographic data were collected. All study echocardiograms were reported by a single independent expert.

### Procedure protocol

Patients underwent invasive coronary angiography according to local protocols. All arteries with a lesion of at least 50% diameter as assessed visually were examined with a pressure wire. Hyperaemia was induced by an intravenous infusion of adenosine, 140 µg/kg/min. The FFR value was measured during maximal stable hyperaemia. The decision to proceed to PCI was at the operators’ discretion, guided by the FFR result.

### Three-dimensional reconstruction of coronary geometry

A three-dimensional (3D) reconstruction of the coronary artery was created offline after the procedure using previously described methods.^7,8^ Two clear orthogonal planes from similar phases of the cardiac cycle, as close to 90° apart as possible, were selected to segment and reconstruct coronary artery luminal geometry. This surface reconstruction was then discretized (meshed) into a finite number of volumetric elements in preparation for computational fluid dynamics (CFD) simulation.

### Coronary microvascular resistance calculation

Personalized CMVR was computed using a 3D CFD simulation with invasively measured proximal and distal pressures at the respective boundaries. CFD simulation was performed to a residual target of 10^−6^ (ANSYS, PA, USA). Using the hydraulic equivalent of Ohm’s law coronary blood flow (*Q*_CFD_) and distal pressure (P_d_) were used to calculate CMVR:

$$\mathrm{CMVR}=\frac{P_{d}}{Q_{\mathrm{CFD}}}$$

### Machine learning (NARMAX) predictive modelling

To identify predictors of CMVR, machine learning (ML) was employed, with a non-linear autoregressive moving average with exogenous inputs (NARMAX) model. Two models were created; the first was based on routinely collected clinical, laboratory, and electrocardiographic data (B), and the second additionally incorporating transthoracic echocardiographic (TTE) data (C). In order to identify the most important variables, a comprehensive cross-validation procedure was performed (random *k*-folder cross-validation). This technique is well validated and frequently employed in the assessment of ML models.^9^ We firstly detect and determine the most important model terms (either single features/variables or cross-product terms). Following common practice, the data points were randomly split into training (75%) and test (25%) data sets. Such splitting was repeated 100 times producing a total of 100 models. The final model was built incorporating the most frequently selected model terms from the 100 runs. To test the performance of the model, we randomly selected 100 sub-data sets from the original data, with 20% leave out in each sub-data set. The model was then run for each of these sub-data sets. Model performance was assessed by calculating the average correlation coefficient and *R*^2^ from the 100 data sets.

### Angio-fractional flow reserve computation (VIRTUheart™)

Angio-FFR was computed using the CFD-based VIRTUheart™ model of coronary physiology as described previously.^3,7^ Three simulations were run for each case, using a generic CMVR (Model A), the predicted CMVR from NARMAX Model B, and the predicted CMVR from NARMAX Model C.

### Statistical analysis

Data are presented as mean (±standard deviation) and number (percentage) unless stated otherwise. Comparison of the quantitative accuracy of the three models was made by comparing average absolute error, mean bias, and correlation coefficients. Agreement was assessed by creating Bland–Altman plots and limits of agreement were compared. All statistics are carried out using SPSS version 26 (IBM, NY, USA).

## Results

### Baseline characteristics

One hundred and fifty-seven arteries were modelled from 84 patients. The mean age was 64.3 (±10) years, 64 (76%) were males, 52 (62%) had hypertension, and 16 (19%) had Type 2 diabetes mellitus. Baseline clinical and lesion characteristics are demonstrated in *Table 1*.

**Table 1:** Baseline patient characteristics

Patient characteristics	*N* = 84
Age	64.2 (±10)
Male	64 (76%)
Hypertension	52 (62%)
Hypercholesterolaemia	59 (70%)
T2DM	16 (19%)
Smoking status	
Never smoked	32 (38%)
Ex-smoker	44 (52%)
Current smoker	8 (10%)
Previous MI	30 (36%)
PVD	4 (5%)
Lesion characteristics	*N* = 157
Vessel	
LAD	76 (48%)
LCX	29 (18%)
RCA	35 (22%)
Dx	13 (8.3%)
OM	2 (1.3%)
Intermediate	2 (1.3%)
FFR	0.79 (±0.15)
Myocardial jeopardy index (%)	32 (±15)

Dx, diagonal artery; LAD, left anterior descending artery; LCX, left circumflex artery; MI, myocardial infarction; OM = obtuse marginal artery; PVD, peripheral vascular disease; RCA, right coronary artery; T2DM, Type 2 diabetes mellitus.

### Prediction of coronary microvascular resistance

Coronary microvascular resistance was successfully computed in all arteries. Mean CMVR was 10.1E+9 (±10.7) Pa/m^3^/s. For Model B (basic clinical and angiographic data), 16 terms were identified as being predictive of CMVR. The top five parameters identified by the model were outlet diameter, vessel (left anterior descending artery, right coronary artery, left circumflex artery, or a major branch), myocardial jeopardy index (MJI), Duke angiographic jeopardy score, and inlet diameter. When this personalized model was applied, the correlation coefficient and *R*^2^ were 0.80 and 0.63, respectively. For Model C, additionally incorporating TTE data, 18 terms were identified and used to build the final model. The top five parameters identified by the model were outlet diameter, end-diastolic intraventricular septal wall thickness, vessel, left-ventricular mass, and minimum lumen diameter. The average correlation coefficient and *R*^2^ were 0.84 and 0.69, respectively.

### Effect of personalization upon angio-fractional flow reserve computation

**Figure 1:**
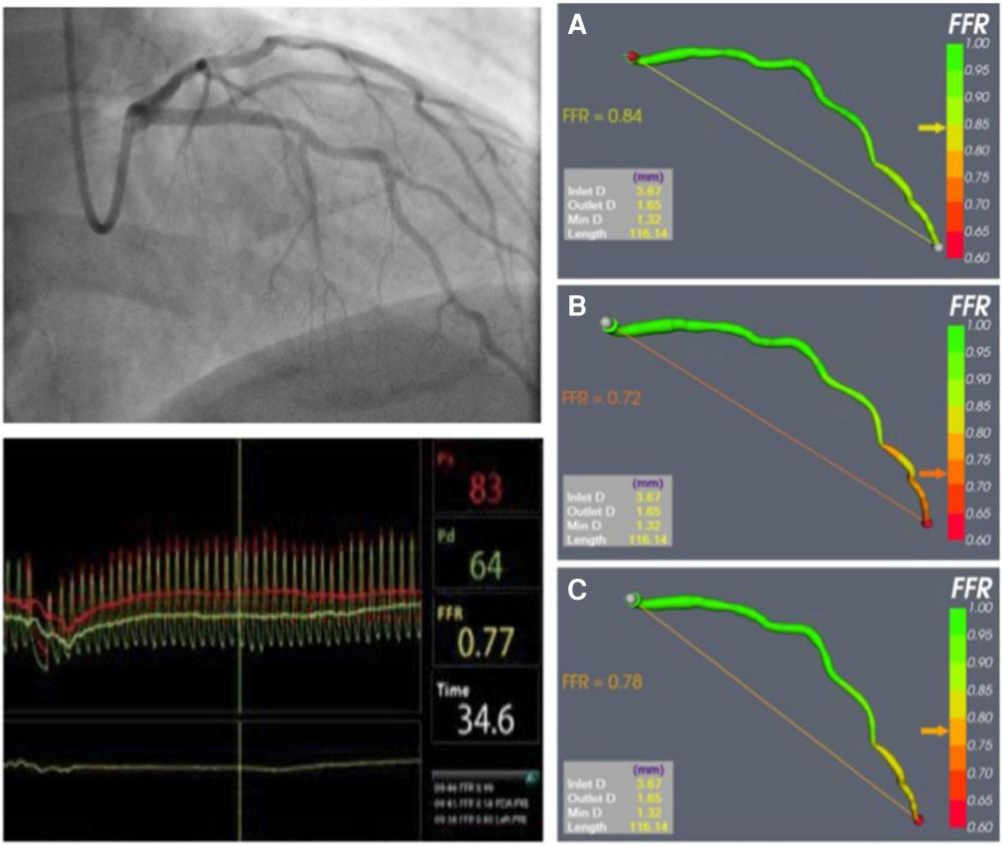
An illustrative example of angio-fractional flow reserve (VIRTUheart™). Coronary angiography revealed disease in the left anterior descending artery (top left). Invasive fractional flow reserve was 0.77 (bottom left). Results from angio-fractional flow reserve modelling are shown on the right-hand panels. Accuracy improved with the personalized models (B and C, angio-fractional flow reserve 0.72 and 0.77, respectively) compared with the generic Model A (angio-fractional flow reserve 0.84).

**Figure 2:**
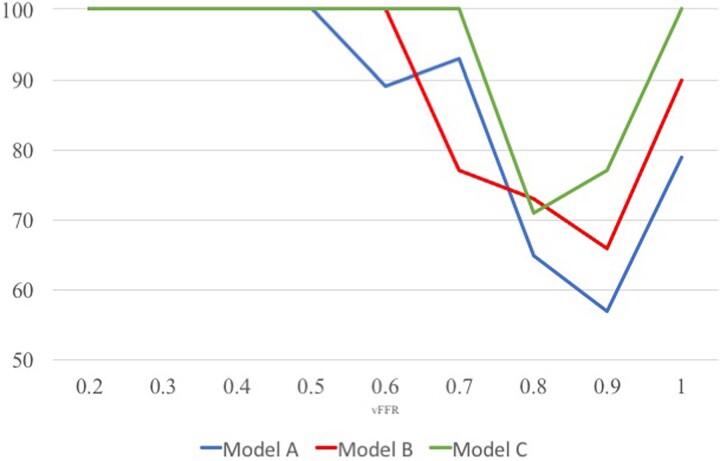
Diagnostic accuracy of each angio-fractional flow reserve models (A–C) across the range of angio-fractional flow reserve values. Diagnostic accuracy (ability to predict fractional flow reserve < or >0.80) for each model was assessed and compared. The blue, red, and green lines reveal the diagnostic accuracy of Models A–C, respectively, across the range of angio-fractional flow reserve values. Accuracy is lowest around the 0.80 diagnostic threshold and highest with the personalized models (B and C).

**Figure 3:**
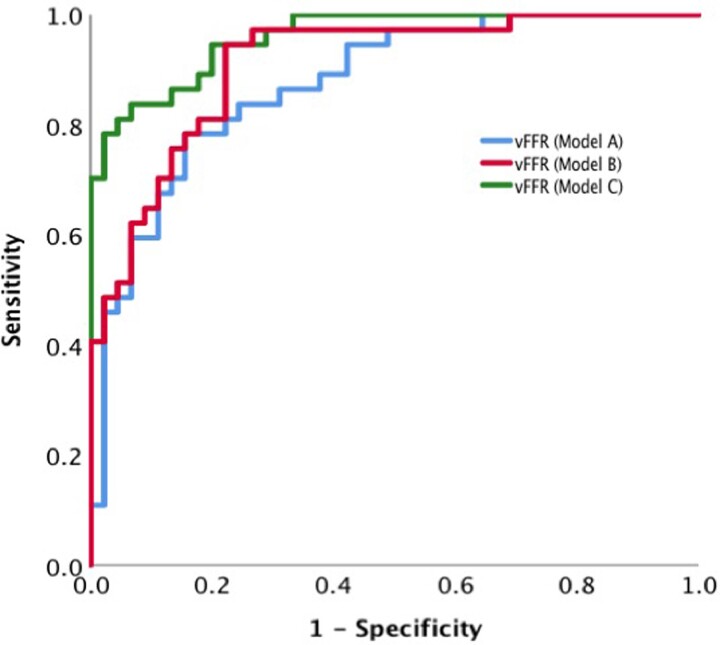
Comparison of receiver-operating characteristic for the three models of angio-fractional flow reserve (A–C). Receiver-operating characteristic curves were created for each model (A–C) and compared. The area under the curve was 0.87, 0.91, and 0.96, respectively. vFFR, virtual (angio-)fractional flow reserve.

**Figure 4:**
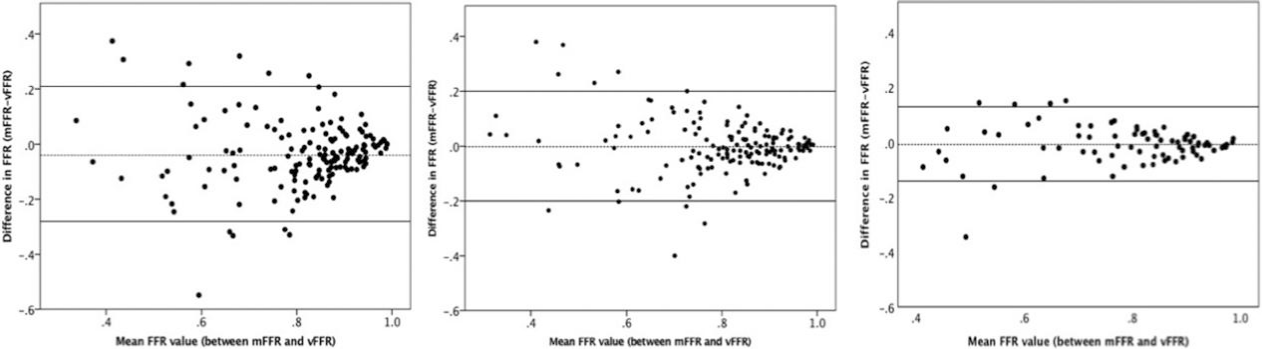
Bland–Altman plots demonstrating agreeability between angio-fractional flow reserve and fractional flow reserve for all three models. Bland–Altman plots demonstrating the difference between measured fractional flow reserve and virtual (angio-)fractional flow reserve plotted against the mean value using the generic coronary microvascular resistance model (left), Model A (centre), and Model B (right). The two dark lines represent the limits of agreement 2 SD above and below the mean delta. mFFR, measured fractional flow reserve; vFFR, virtual (angio-)fractional flow reserve; CMVR, coronary microvascular resistance.

Using personalized CMVR significantly improved the diagnostic and quantitative accuracy of angio-FFR (VIRTUheart™). An example case in shown in *Figure 1*. Diagnostic accuracy improved with model personalization from 73% for Model A to 81% with Model B and 89% with Model C (*P* < 0.001). The diagnostic accuracy of each model across the range of angio-FFR values is demonstrated in *Figure 2*. On receiver-operator characteristic curve analysis, area under the curve was 0.88, 0.91, and 0.96 for Models A–C, respectively (*Figure 3*). Average absolute error reduced with personalization from FFR ±0.10 for Model A, to FFR ±0.07 with Model B, and FFR ±0.05 with Model C (*P* < 0.001). A full breakdown of results is shown in *Table 2*. Bland–Altman plots are shown in *Figure 4*.

**Table 2:** Effect of coronary microvascular resistance personalization upon angio-fractional flow reserve (VIRTUheart™) accuracy

	Angio-FFR (generic, A)	Angio-FFR (NARMAX, B)	Angio-FFR (NARMAX, C)	*P*-value
	*n* = 157	*n* = 157	*n* = 80	
Mean delta (bias)	−0.036 (±0.12)	−0.0018 (±0.10)	−0.007 (±0.07)	0.30
Limits of agreement	−0.28 to 0.21	−0.20 to 0.20	−0.15 to 0.13	
Average error	0.095 ± 0.088	0.069 ± 0.074	0.05 (±0.05)	<0.001
Correlation	0.65	0.75	0.79	0.02
Diagnostic accuracy	73%	81%	89%	0.005
Sensitivity	54%	71%	86%	<0.001
Specificity	86%	89%	91%	0.27
PPV	73%	83%	89%	0.005
NPV	72%	80%	89%	0.003

NPV, negative predictive value; PPV, positive predictive value.

## Discussion

We have demonstrated that CMVR can be estimated from routinely collected clinical data. Moreover, personalizing angio-FFR computation by incorporating model-predicted CMVR significantly improved diagnostic and quantitative accuracy. The addition of TTE data to the model further improved angio-FFR accuracy.

### Inaccuracy in angio-fractional flow reserve computation

Coronary angiogram-derived FFR has emerged as a non-invasive alternative to pressure wire based FFR. Since its first description by Morris *et al.* in 2013,^3^ the computational technology has advanced, processing times have been reduced, and a number of manufacturers now offer commercial angio-FFR solutions that are beginning to be incorporated into clinical practice.^2,4,10–13–14^ The way in which the accuracy of these models is reported varies. Most studies primarily quote the diagnostic accuracy (ability to predict FFR  ≤ or >0.80); which is largely similar between models (∼85–90%). Quantitative or ‘absolute’ accuracy (the ability to predict the actual FFR value) is less well reported and, when it is, is relatively poor, with 95% limits of agreement in the order of ±0.14 for most models (*Table 3*).^11–14^ Although methods vary slightly between groups, the overall principles remain the same. Angio-FFR is derived by applying a mathematical solution of flow, based upon the laws of fluid dynamics, to a geometric reconstruction of coronary anatomy that is derived from the coronary angiogram. Potential sources of error can arise due to the accuracy of the 3D reconstruction (not considered in this study, but generally applicable to all model systems) and to assumptions made when defining the boundary conditions.

**Table 3:** Comparison of the accuracy of available angio-fractional flow reserve solutions

	Angio-FFR (NARMAX)	Cathworks^11^	PIE medical CAAS vFFR^12^	Medis QFR^13^	caFFR E^14^
	VIRTUheart™				
No. of vessels	80	319	334	969	328
Mean delta (bias)	−0.007 (±0.07)	—	0.002 (±0.06)	0.009 (±0.07)	−0.002 (±0.05)
Limits of agreement	−0.15 to 0.13	−0.14 to 0.12	−0.12 to 0.12	−0.12 to 0.14	−0.10 to 0.09
Average error	0.05 (±0.05)	—		—	
Correlation	0.79	0.80	0.74	0.80	0.89
Diagnostic accuracy	89%	92%	90%		95.7%
Sensitivity	86%	94%	81%	84%	90.4%
Specificity	91%	91%	95%	88%	98.6%
PPV	89%	89%	90%	80%	97.2%
NPV	89%	95%	90%	95%	95%

NPV, negative predictive value; PPV, positive predictive value.

### Defining the boundary conditions

The boundary conditions are the physical conditions at each of the boundaries of the model. For a coronary arterial model, there are three boundaries: the inlet, the vessel wall, and the outlet. For patients undergoing invasive angiography, the inlet boundary is known precisely (proximal aortic pressure). The vessel wall is typically modelled as a rigid wall. Although this is not physiologically accurate, this method is widely accepted in coronary circulation models, because small variations in the vessel calibre are averaged out over the cardiac cycle.^15^ The distal (outlet) boundary presents the greatest challenge. This boundary is the distal CMVR, which regulates coronary blood flow and is known to vary from patient to patient in healthy and diseased states; yet there is currently no way to measure it, without invasive instrumentation. Therefore, current models rely upon assumptions and generalizations when defining their models. The Medis QFR® model (Medis Medical Imaging, Leiden, The Netherlands) uses TIMI frame counting to estimate flow in one version of the model,^2^ the PIE medical CAAS vFFR system (PIE Medical Imaging, Maastricht, The Netherlands) estimates hyperaemic blood flow empirically from clinical data^4^ and the Cathworks FFR angio™ system (Cathworks Ltd, Israel) uses scaling laws to estimate the microcirculatory bed resistance.^10^ In VIRTUheart™, the modelling system used in this study, a generic CMVR value can be applied to all patients^3^ or, as in this study, it can be adapted by incorporating a panel of personalized parameters. The assumptions regarding the boundary conditions are the greatest source of error, as shown in a previous study.^6^ We therefore postulated that by personalizing CMVR, we could significantly reduce error in angio-FFR computation. In the current study, personalization increased the diagnostic accuracy of VIRTUheart™ angio-vFFR from 73 to 89% and halved the average absolute error (from 0.10 to 0.05). This is the first time that angio-FFR has been personalized to this extent.

### Predictors of coronary microvascular resistance

The most important predictors of CMVR were arterial outlet diameter, MJI, vessel, inter-ventricular septal wall thickness, and left-ventricular mass. These are clearly all related to subtended myocardial mass, which is in turn related to CMVR. Arterial outlet diameter has a direct relationship with CMVR that has previously been described.^16^ Based upon the hydraulic equivalent of Ohm’s law, CMVR can be calculated as the ratio of distal pressure and coronary blood flow. Thus, CMVR is related inversely to flow, which is in turn related to vessel size. The relationship between vessel size and flow rate was first proposed by Murray, who stated that flow is proportional to vessel diameter to an exponent of an empirically derived constant (*k*).^16^ This relationship can be understood by considering the Hagen–Poiseuille law which states that where *Q* = flow, Δ*P* = pressure drop, *r* = vessel radius, *h* = viscosity, and *l* = vessel length:

Q=ΔPπr4/8hl.

The MJI provides an estimation of the subtended myocardial mass, based upon angiographic coronary anatomy.^17^ Each coronary vessel is assigned a score from 0 to 3 depending on its size (3 = a large vessel covering >2/3 of the distance from base to apex, 2 = a medium-sized vessel covering between 1/3 and 2/3 distance from base to apex, 1 = a small vessel covering <1/3 distance base to apex and 0 = an insignificant vessel). The MJI is then calculated as the total score of all the branches distal to the lesion being studied as a proportion of the total score of all vessels. Therefore, a 70% lesion located at the left main coronary artery will have a substantially greater MJI than a 70% lesion in a distal branch. In the present study, MJI was inversely related to CMVR. This is because, at each branching point, flow decreases and the driving pressure remains relatively constant. In accordance with our findings, a previous study demonstrated an inverse relationship between MJI and the index of microvascular resistance measured at invasive coronary angiography.^18^ TTE-derived inter-ventricular septal wall thickness and left-ventricular mass provide a direct assessment of total myocardial mass and ventricular wall hypertrophy. Total coronary flow is proportional to myocardial mass; therefore, it is unsurprising that we demonstrated an inverse relationship between these parameters and CMVR.

### Personalizing angio-fractional flow reserve prediction

Simple personalization of the coronary arterial distal boundary based upon estimates of outlet vessel diameter and left ventricular mass has previously been described in the derivation of FFR-CT. In the Heartflow™ FFR-CT model, outlet pressure is predicted from the diameters of the coronary outlets and the myocardium subtended (estimated from CT-derived ventricular volume quantification).^19^ As this model is based upon CT, these data are more readily available. Ours is the first description of this level of personalization of angio-vFFR models. Moreover, we have extended our analysis to incorporate other clinical parameters that influence CMVR beyond these two measures.

Importantly, we have produced two models; one with, and one without, TTE data. Incorporation of TTE data significantly improved the accuracy of our model as it can provide an accurate estimation of LV mass and septal wall hypertrophy. However, not all patients attending the catheter laboratory will have had a TTE as part of their clinical routine. We therefore wanted to produce a simplified model, that, even without TTE, could provide an estimation of CMVR, improving angio-vFFR accuracy. The operator can then choose the appropriate model based upon the data available to them at the time. Equally, where TTE is not available, a quick focused scan could be performed to obtain the key parameters.

### Limitations

The sample size of this study was modest, although it was larger than similar validation studies. Many of the model parameters, in particular those derived from TTE, are likely to be subject to a degree of inter-observer variability. The impact of this on model accuracy was not assessed as part of the current study. Further work will be required to determine the applicability of our model, which was based on data from a single centre. External validation will also be required. Some data were not available, and therefore, missing imputation was used. Personalization was based upon the VIRTUheart™ model of angio-FFR. Whether our model would be applicable to other angio-FFR models remains to be determined. When compared with other commercial solutions, personalized angio-FFR (VIRTUheart™) performed similarly (*Table 3*); however, caution is required when comparing diagnostic accuracies because each modality was validated on a different patient population. This study was not designed to determine the superiority of the VIRTUheart™ model over other commercial solutions.

## Conclusions

The accuracy of angiography-derived FFR is dependent upon CMVR estimation. This parameter can be personalized based upon routinely collected clinical data, leading to a significant reduction in error. This effect is further enhanced when TTE data are additionally incorporated.

## Data Availability

The data underlying this article will be shared on reasonable request to the corresponding author.

## Lead author biography

Rebecca C. Gosling is an NIHR clinical lecturer in cardiology at the University of Sheffield and honorary cardiology registrar at the Sheffield teaching hospitals NHS foundation trust. Her academic interests are in computational modelling and its application to cardiac imaging techniques with a focus on developing personalized medicine approaches.

## Impact on daily practice

Using ML techniques, it is possible to predict vessel-specific CMVR from routinely collected clinical and TTE data. When personalized, CMVR values are incorporated into the angio-FFR model and accuracy is significantly improved. This level of personalization can be achieved with and without the incorporation of TTE parameters, the latter providing the highest accuracy.
